# Visualization Methods for Uterine Sentinel Lymph Nodes in Early-Stage Endometrial Carcinoma: A Comparative Analysis

**DOI:** 10.3390/diagnostics14050552

**Published:** 2024-03-05

**Authors:** Linas Andreika, Karolina Vankevičienė, Diana Ramašauskaitė, Vilius Rudaitis

**Affiliations:** 1 Clinic of Obstetrics and Gynecology, Institute of Clinical Medicine, Faculty of Medicine, Vilnius University, M. K. Čiurlionio Str. 21/27, LT-03101 Vilnius, Lithuania; diana.ramasauskaite@mf.vu.lt (D.R.); vilius.rudaitis@mf.vu.lt (V.R.); 2 Faculty of Medicine, Vilnius University, M. K. Čiurlionio Str. 21/27, LT-03101 Vilnius, Lithuania

**Keywords:** endometrial cancer, endometrial carcinoma, sentinel lymph node mapping, sentinel lymph node biopsy

## Abstract

Background: Sentinel lymph node (SLN) biopsy in early-stage endometrial cancer is recommended over systematic lymphadenectomy due to reduced morbidity and comparable detection rates. The main objective of this study was to compare the overall and bilateral detection rates of SLN in early-stage endometrial cancer using three techniques. Methods: a prospective cohort study was designed to detect the difference in SLN detection rate in three cohorts: Indocyanine green (ICG), methylene blue (MB), and tracer combination (ICG + MB). Mapping characteristics, detection rate, number of SLNs, and positive SLNs of the three cohorts were compared. Results: A total of 99 patients were enrolled. A total of 109 SLN sites with 164 lymph nodes were detected. No differences were found between the three cohorts in terms of age, BMI, tumor diameter, or other histologic characteristics. The overall SLN detection rate (DR) was 54.3% in the MB group, 72.7% in ICG, and 80.6% in the ICG-MB group. Bilateral DR was 22.9%, 39.4%, and 54.8% in groups, respectively, with the MB method yielding significantly inferior results. Conclusions: The ICG-MB group demonstrated superior overall and bilateral detection rates, but a significant difference was found only in the MB cohort. Combining tracer agents can enhance the accuracy of SLN identification in initial-stage endometrial cancer without additional risk to the patient.

## 1. Introduction

According to GLOBOCAN, in 2020, there were 417,367 newly diagnosed cases of corpus uteri cancer, which caused 97,370 deaths. The age-standardized incidence rates worldwide reached 8.7 cases per 100,000. High-income areas such as North America, Central and Eastern Europe, and Northern Europe had the highest rates, reaching 21.1, 20.2, and 16.4 cases per 100,000, respectively. In Lithuania, uterine cancer is ranked third among the top five most frequently diagnosed female cancers, following breast and colorectal cancers [1].

The majority of uterine cancers are of epithelial origin, with endometrioid adenocarcinomas (EC) being the most common subtype. Sarcomas of mesenchymal origin account for less than 9% of diagnoses. Among EC, around 80% are classified as type I and histologically belong to the endometrioid type, with the majority diagnosed as low grade (International Federation of Gynecology and Obstetrics (FIGO) grade 1 or 2) [2]. This type of EC usually manifests with early symptoms such as postmenopausal bleeding or abnormal uterine bleeding in premenopausal women, thus generally resulting in early diagnosis with a good prognosis and high survival rates. Another 20% are classified as type II malignancies and histologically belong to clear cell EC, serous EC, mixed cell EC, carcinosarcoma, and undifferentiated/dedifferentiated carcinoma types. More than half of them are high-grade (FIGO grade 3) tumors, which are independent of estrogen exposure, poorly differentiated, with a higher risk of metastases recurrence, and have an overall poor prognosis [3,4]. 

According to the European Society of Gynecological Oncology (ESGO), the European Society for Radiotherapy and Oncology (ESTRO), and the European Society of Pathology (ESP) ESGO/ESTRO/ESP guidelines for the management of patients with endometrial carcinoma, EC is categorized into prognostic risk groups based on histopathological type, grade, myometrial invasion, lymphovascular invasion, and molecular type, if known. Clinical decisions, the extent of surgical treatment, and adjuvant treatment are determined based on those prognostic risk groups [5].

In early-stage endometrial cancer, the primary treatment is surgery, including a total hysterectomy with bilateral salpingoophorectomy [6]. In EC, lymph node metastasis is the most common pattern of extrauterine spread. Lymph node evaluation is incorporated into surgical EC management and is considered to be one of the most crucial prognostic factors and the strongest predictor of disease recurrence [7]. However, only up to 10% of all patients in early-stage EC will be diagnosed with lymph node metastasis. Thus, the best method for lymph node assessment remains controversial, as the majority of patients who undergo the classical approach of systematic lymphadenectomy have no metastases but suffer the consequences of lymph node removal [8]. 

Today’s modern approach to sentinel lymph node biopsy (SLNB) dates back to 1992 when Morton et al. reshaped the understanding of cancer dissemination via lymphatics. They were the first to propose that metastatic melanoma systematically progresses from the sentinel lymph node (SLN) through the chain of second or third-tier lymph nodes. The main idea behind SLNB is identifying and doing a biopsy of the first lymph node or nodes that drain the primary tumor. If the SLN is free of cancer cells, it is assumed that the cancer has not spread to other lymph nodes [9].

There is a lack of scientific evidence that systematic lymphadenectomy reduces the risk of death or disease recurrence in stage I endometrial carcinoma compared to no lymphadenectomy [10]. Currently, there is a Grade A ESGO/ESTRO/ESP recommendation in lymph node staging against systematic lymphadenectomy, including pelvic and para-aortic infrarenal lymph node dissection for low-risk/intermediate-risk prognostic group EC. Instead, SLNB is recommended in these groups [5].

In cases where the risk of lymph node involvement is low, the use of SLNB aligns with efforts to minimize the morbidity associated with systematic lymphadenectomy and assess the suitability of adjuvant treatment while preserving accurate staging information. SLNB approach aims to reduce the potential intra- and postoperative complications associated with systematic lymphadenectomy, including intraoperative bleeding, injury to surrounding tissues and organs, lymphocele, and lymphedema [11].

Tracer dyes such as blue dye, indocyanine green (ICG), and technetium-99m are used to detect SLN in either or each hemipelvis. The SLN detection rate in general is reported to be 86.9%, and the bilateral detection rate is 65.4%; the detection rate varies among the tracer dyes, ranging from 77.8% using blue dye alone to 100% using ICG combined with technetium-99m. It is reported that detection rates with tracer dye combinations, for example, using ICG and blue dye, may be higher compared to using one tracer dye alone [12]. 

Although blue dyes have predominantly been used for SLN detection in Lithuania, in light of recent recommendations for clinical practice, ICG was introduced for SLN visualization as well. The main objective of this study was to compare the overall and bilateral detection rates for SLNB in early-stage endometrial cancer using three techniques: ICG alone, MB alone, and the two dyes in combination. Additionally, the study aimed to supplement existing scientific data with findings obtained from a tertiary-level university hospital in Lithuania. After completing the research, in order to compare the key findings of this study with others, an advanced search on PubMed was conducted incorporating such keywords as “endometrial cancer”; “uterine cancer”; “sentinel lymph node”; “methylene blue”; “blue dye”; “indocyanine green”. The studies included for the comparison were limited to those written in English and published in the last 10 years. 

## 2. Materials and Methods

### 2.1. Patient Population

From April 2020 to October 2023, 99 patients over 18 who were diagnosed with endometrial cancer (2009 FIGO classification stage I, any grade or histology) were enrolled in this study. Excluded from the study were patients with radiological evidence of extrauterine disease; those with a history of previous bilateral pelvic or para-aortic lymphadenectomy or radiotherapy due to other malignancies; those not suitable to undergo surgery with curative intention; those allergic to iodine or methylene blue (MB) dye; and those who did not provide written consent to be enrolled in the study. Enrolled patients were assigned to three study groups according to used tracers: MB, ICG, and ICG-MB. To avoid patient selection bias, patients were assigned to one of the study groups using permutated blocks and stratified randomization techniques. Tracers were assigned to blocks so that they were random in order but that the desired allocation proportions were achieved evenly within each block. In this study, 3 blocks of 30 patients were established, ensuring a similar number of subjects in each group at all times. In each block, 10 patients were assigned to the MB, 10 to the ICG, and 10 to the ICG-MB group. At the time of randomization, we applied stratification by characteristics that were measurable and could have had an impact on the research results, such as patient age, BMI, and tumor histology; therefore, all three patient groups had no significant differences. Data were collected prospectively and maintained in a Microsoft Excel-based electronic database (Microsoft, Redmond, WA, USA). The study was approved by the Vilnius Regional Biomedicine Research Ethics Committee (permit number 2020/3-1206-691 31 March 2020) All patients enrolled in the study gave written and informed consent. 

### 2.2. SLN Mapping Procedures

In September 2019, a new uterine cancer protocol was introduced in Vilnius University Hospital Santaros Clinics Gynecology Department, approving SLN detection as a possibility for initial-stage endometrial cancer patients undergoing laparoscopic total hysterectomy.

In February 2020, fluorescent detection with ICG was initiated using the Olympus Visera Elite II OTV-S300 video system, CH-S200-XZ-EB camera, ESG-400 and USG-400 generators, and CLV-S200-IR light source (Olympus Corporation, Tokyo, Japan) for minimally invasive procedures.

Literature findings and early experiences showed the possibility of combining two tracers to improve detection rates, which were lower than expected when using either MB or ICG. Prior to the study, we found that the best results were achieved using a 4-site injection technique instead of 2. Therefore, we adopted it as a standard for our study. The 12 o’clock injection site was not utilized due to a high chance of staining the vesicouterine plica, which complicates the bladder dissection.

The ICG concentration used in this study was 1.25 mg/mL. For each patient, a 25 mg vial with ICG powder (Verdye, Diagnostic Green GmbH, Munich, Germany) was diluted with 20 mL of sterile 0.9% NaCl saline. A total of 8 mL of the ICG solution was injected into the cervix alone, divided into the 2, 5, 7, and 10 o’clock positions. One milliliter of the ICG solution was injected into the submucosal layer of the cervix and another 1 mL into the stroma, with penetration to a depth of up to 1 cm. The MB concentration used was 0.25%, 10 mg/mL (prepared by our hospital pharmacy), with 8 mL injected into the cervix alone using the same technique as for ICG. In the combination group, 8 mL of MB was injected, followed by a 5-min pause, and then 8 mL of ICG was injected at the same injection points. 

The Hohl’s uterine manipulator would not be inserted until SLNs were detected intraoperatively by the laparoscopic fluorescence camera in the ICG and combination group or visually in the MB group.

In the event of unsuccessful SLN detection, patients in the low-risk group did not undergo systematic lymphadenectomy unless suspiciously enlarged lymph nodes were found. Patients in the intermediate and high-risk groups underwent SLN biopsy and a comprehensive pelvic and aortic lymphadenectomy. Some patients in the intermediate or high-risk groups were deemed unsuitable for aortic lymphadenectomy due to comorbidities; therefore, only a bilateral pelvic lymphadenectomy was performed. Following ESGO guidelines, frozen sections were not performed during operations.

### 2.3. Pathologic Evaluation

All SLNs were sent for pathologic evaluation separately from non-SLN and paraffin-embedded as per standard protocol. Stains with hematoxylin and eosin (H&E) were performed. Ultrastaging was not conducted, as it is expensive and not routinely performed in our pathology center. Pathological disease staging was conducted in accordance with the 2009 FIGO classification [13] after obtaining definitive histology results.

### 2.4. Data Analysis

Absolute and percentage frequencies were employed to describe categorical items, while mean or median values, standard deviation, and range were assessed for continuous characteristics. The normality of quantitative variable distributions was tested using a goodness-of-fit test (Shapiro–Wilk). For the analysis of continuous-categorical variables, an ANOVA permutation test was applied. Fisher’s exact test was used for the analysis of categorical-categorical variables. A significance level of *p* < 0.05 was adopted. The statistical analysis was conducted using the pandas Python module and Rpy2 (The Python Software Foundation, Wilmington, DE, USA). Fisher’s Exact Test was conducted using Rpy2: a Python interface to the R language. All other statistical tests came from Python’s module from the SciPy library.

## 3. Results

From April 2020 to October 2023, 99 patients diagnosed with initial-stage endometrial cancer (any grade or histology) were enrolled in this study. Among them, 35 patients (35.4%) were assigned to the MB group, 33 (33.3%) to the ICG group, and 31 (31.3%) to the ICG-MB combination group.

### 3.1. Patient Characteristics

The mean age of the general cohort was 62.9 years, ranging from 35 to 83 years. The mean body mass index (BMI) was 32 kg/m^2^, ranging from 18 to 50 kg/m^2^. The vast majority of the patients (89%) were diagnosed with EC, and 87.9% had grades 1–2. The mean maximum diameter of the tumor was 27.5 mm, ranging from 1 to 85 mm. A total of 62.6% of the patients were diagnosed with FIGO stage IA, 27.3% with stage IB, and 10.1% were diagnosed with advanced II-III stages (8 patients due to lymph node metastatic involvement, 1 due to metastasis on the pelvic sidewall, 1 due to tumor invasion into the stroma of the cervix). Lymphovascular invasion (LVI) was found in 18.2% of the patients (Table 1).

**Figure 1:**
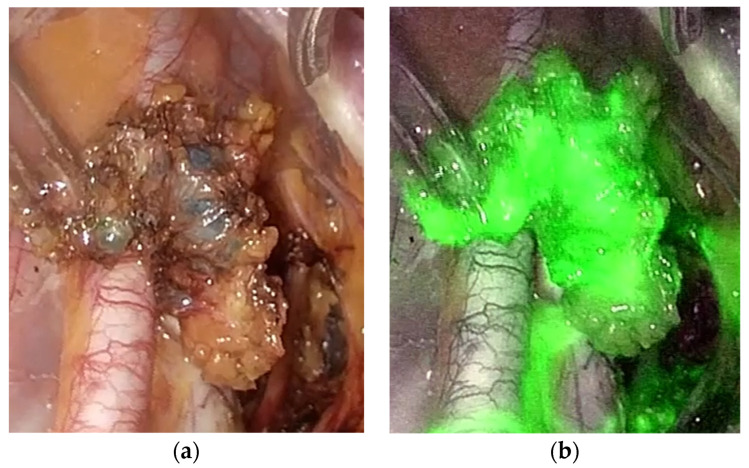
(**a**) Image of uterine SLN traced with MB; (**b**) Image of uterine SLN traced with ICG (in ICG-MB combination). Both images were observed during laparoscopic surgery.

No differences were found between the three cohorts in terms of age, BMI, tumor diameter, or other histologic characteristics, except lymphovascular invasion, where the ICG-MB group had no LVI cases (Table 1). LVI differences were accidental due to block randomization and the lack of possibility of applying stratification, as the LVI could not have been predicted prior to the operation. A minimally invasive approach was preferred in all cases (Figure 1). There were no cases of allergic reactions to any of the tracers, although we recorded two cases of brief saturation decrease associated with MB injection, which resolved without any additional measures.

### 3.2. Comparison of SLN Mapping Modalities

The overall detection rate (DR) of SLN mapping was 54.3% in the MB group, 72.7% in the ICG group, and 80.6% in the ICG-MB group. The bilateral DR was 22.9%, 39.4%, and 54.8% in the MB, ICG, and ICG-MB groups, respectively. The aortic DR was 2.9%, 6.1%, and 9.7%, respectively (Table 2).

Despite apparent differences between the groups, a statistically significant difference was found only between the MB and ICG-MB cohorts (overall DR *p* = 0.035; bilateral DR *p* = 0.011). No statistically significant differences were observed between the ICG and ICG-MB groups in terms of overall detection rate (*p* = 0.556) and bilateral detection rate (*p* = 0.316). Notably, the ICG cohort did not demonstrate statistically significant differences when compared to the MB group (overall DR *p* = 0.137; bilateral DR *p* = 0.191). Even though ICG was the primary tracer in the ICG-MB combination group, being detected in all 25 cases (80.6%) where SLNs were identified, MB was visible in only 15 cases (48.4%) (*p* = 0.001) (Table 3).

MB proved to be useful in two patients when ICG stained the entire internal iliac region, and SLNs were challenging to detect, relying solely on ICG.

**Figure 2:**
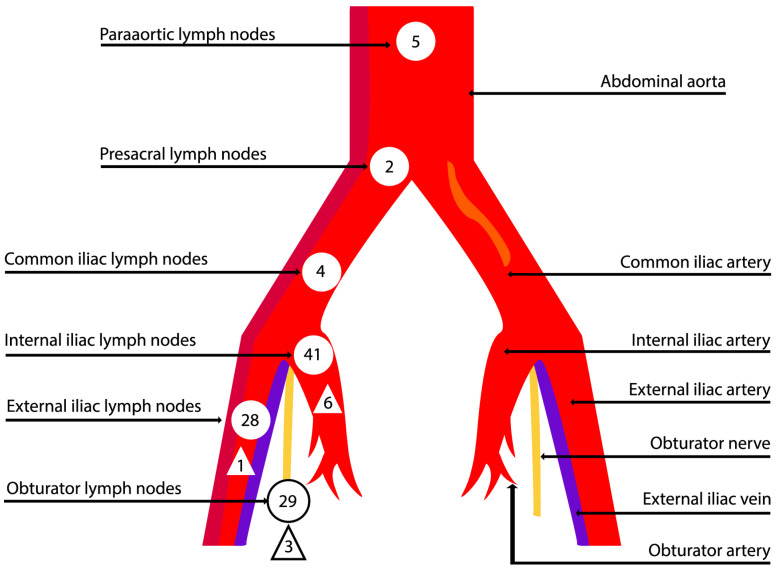
Sites for detecting SLNs (round shape) and metastases (triangular shape).

A total of 109 SLN sites with 164 lymph nodes were detected, most commonly in the internal iliac region (37.6%) (Table 4) (Figure 2).

The median number of SLNs detected per patient was 1 (ranging from 0 to 4) in the MB group, 2 (ranging from 0 to 8) in the ICG group, and 2 (ranging from 0 to 7) in the ICG-MB group (*p* = 0.222). The mean size of the SLN was 18.7 ± 9.7 mm (ranging from 4 to 37 mm), 13.2 ± 5.2 mm (ranging from 6 to 23 mm), and 14.1 ± 6.7 mm (ranging from 5 to 32 mm), respectively (p = 0.964). Out of the total, seven patients (7.1%) had positive SLNs: 4 (11.4%) in the MB group, 3 (9.1%) in the ICG group, and none in the ICG-MB group (*p* = 0.017). The occurrence of “empty node packets” in the ICG group was 5.9%, while both the MB and ICG-MB groups showed none (0%). However, these differences did not reach statistical significance (*p* = 0.075) (Table 5).

Due to the fact that only 11 patients (11.1%) underwent a complete pelvic and aortic lymphadenectomy, the sensitivity and negative predictive value of these SLN detection techniques could not be calculated in our study, which is one of our study’s limitations.

## 4. Discussion

The use of MB is characterized by cost-effectiveness, non-toxicity, and prolonged residence times in lymph nodes. It is easy to obtain and does not require extra equipment. Thus, it is available for both open and minimally invasive surgeries. Because of its low cost and accessibility, it is suitable for specialists in low-resource countries, where minimally invasive surgery and other tracers are unavailable. Despite lower than other tracer detection rates, it can still serve as a practical alternative to not using any tracer at all [14]. By employing MB in low and intermediate-risk group patients, we gained valuable experience and found it to be useful: if the SLNs were found, intermediate-risk group patients did not undergo systematic lymphadenectomy.

The primary drawback of MB became evident with its dark blue color. As it infiltrated the paracervical tissues, tissue dissection became challenging since the free tissue spaces became nearly invisible. In scientific research, several disadvantages are associated with MB beyond the discoloration of the operative field. Other drawbacks include allergic reactions to the blue dye, such as urticaria, skin rashes, erythema, blue hives, cardiovascular collapse, and anaphylactic shock. Additional concerns involve temporary skin tattooing, the presence of blue-colored urine for up to 24 h following administration, and a fictitious drop in intraoperative oxygen saturation measured by pulse oximetry [15]. While we did not encounter complications related to tissue coloring during the operation, nor did we observe any serious side effects, a drop in saturation was noted in several cases. This occurrence was resolved without the need for additional measures.

In comparison to MB, ICG is significantly more expensive, and additional equipment is required for the visualization of the tracer [16]. Nevertheless, ICG offers the highest detection rate, surpassing not only MB but also the technetium-99m-MB combination, which was once regarded as the gold standard in SLN mapping before the era of ICG [17]. ICG is not only a reliable method for detecting SLNs but is also feasible, safe, and time-efficient [18]. Another significant advantage of ICG over MB is that the near-infrared light is invisible to the operator. As a result of this characteristic, the appearance of the operative field remains unaltered, contrasting with the potential alteration observed when MB is used [17]. Additionally, there is evidence indicating that ICG is more effective than MB in detecting SLNs in obese patients, given its ability to be identified without the need for tissue dissection. However, our study did not observe such an association. For instance, a study conducted by Eriksson et al. confirmed that with an increase in BMI, the rates of overall and bilateral SLN mapping decrease when using either ICG or blue dye. However, they found that mapping rates were significantly improved with the use of ICG in obese and morbidly obese patients. The authors recommended that ICG should be the dye of choice for this specific patient population [19]. Given that many patients with endometrial cancer are overweight, it becomes imperative to carefully consider the method chosen for identifying SLNs. Despite the evident advantages of ICG, our study did not yield significant differences between ICG and MB (*p* = 0.137), likely attributed to the limited number of patients at the current stage of the study.

Our study showed that the least effective method of visualizing SLNs was MB alone. The trend is also notable in the systematic literature review with a meta-analysis conducted by Lin et al., involving 44 studies in the analysis of SLN mapping using blue dye alone, radiotracer dye with blue dye, and ICG. This research revealed that when mapping was performed exclusively with the blue dye, the pooled detection rate was observed to be relatively low [15].

Then, there is a possibility of a dye combination. Combining blue dye (isosulfan blue) and a radioactive tracer (technetium-99m) for SLN mapping in breast cancer surgery began in the early 1990s. This approach marked a significant advancement in enhancing the accuracy of SLN detection [20]. Systematic literature review with meta-analysis by Nagar et al. involved 33 studies comparing the characteristics of not only blue dye, ICG, and technetium-99m but also their combinations. The research suggests that the diagnostic test accuracy for SLNB using either ICG alone or a combination of a dye (blue or ICG) with technetium-99m has a high sensitivity. Moreover, the detection rates using a combination of dyes may be higher [12]. The combination of ICG and MB was assessed in a prospective study conducted by Holloway et al. Findings revealed that this combination detected more SLNs and more lymph node metastases compared to ICG alone. The evaluation of SLNs with the combination of ICG and MB demonstrated excellent sensitivity for the detection of metastasis, and no safety issues were identified [21]. Our study complements the existing data, highlighting that staining is most efficient when both tracers (ICG-MB) are used in combination. 

Research is currently in progress, including studies involving the combination of ICG and technetium-99m in early-stage endometrial cancer. For instance, in the prospective study conducted by Cabrera et al., which compared Tc-99m-ICG with Tc-99m-blue, the overall detection rate was 93%, and it was not statistically different between the two groups. However, a better bilateral detection rate was observed among Tc-99m-ICG patients [22]. Nevertheless, technetium-99m is linked to disadvantages associated with radioactive colloids. Its primary drawbacks include high costs and producing large quantities of highly radioactive waste. Additionally, it requires preoperative injection and imaging, special intraoperative detection equipment, and the safe handling of a radioactive substance [23,24]. These were the main reasons why we chose not to use technetium-99m in our study.

Although we observed statistically significant differences only between ICG-MB and MB cohorts, SLN DR reached its highest at 80.6% when both dyes were used in the ICG-MB group. The bilateral detection rate, with a statistically significant relationship, was also the highest in the ICG-MB group, reaching 54.8%. The superior staining of lymph nodes achieved through a combination of dyes may be attributed to various factors. Combining dyes may exhibit different ways of penetrating the lymphatic system; also, MB has a slower spread rate, thus staining a smaller area compared to ICG. Therefore, the dyes complement each other and enhance the benefits of using both dyes together based on their distinct properties and coverage. For example, when evaluating the combination method, in half of the cases (16 out of 31), MB did not stain, and SLNs were biopsied only by traces of ICG. Despite ICG being the primary tracer, there were instances where the entire region was stained by it, and SLNs were only detected with the assistance of MB, which stained a smaller area. Furthermore, the ICG cohort had 4 (5.9%) “empty node packets”, whereas, in the other cohorts with MB, none were detected. Similar findings were reported by The FILM trial, which documented the absence of nodal tissue in 5% of the specimens detected with ICG compared to 0% in those detected with MB [25]. Thus, the combination may lead to a more precise distinction of SLNs. 

Regarding the number of SLNs detected by different methods, no statistically significant relationship was found. However, it should be noted that the number of lymph nodes obtained with ICG alone or in combination was higher. The median number of SLNs detected per patient was 1 (ranging from 0 to 4) in the MB group, 2 (ranging from 0 to 8) in the ICG group, and 2 (ranging from 0 to 7) in the ICG-MB group. This observation emphasizes the advantages of ICG. Nevertheless, it cannot be ruled out that MB might be more effective for SLN detection because it tends to stay localized, whereas ICG may flow through all lymphatics and lymph nodes, potentially leading to a higher number of SLNs detected in the ICG group.

In our study, only 7.1% of patients with early-stage EC had positive SLNs. This finding supports prior research, which indicated that in early-stage endometrial carcinoma, only up to 10% of patients have positive SLNs. Our study complements existing data on positive SLNs, suggesting that fewer patients require complete lymphadenectomies [8]. This approach avoids the associated lower quality of life due to possible complications related to systematic lymphadenectomy. It is noteworthy that the majority of patients with positive SLNs were identified in the MB cohort (4), in contrast to the ICG (3) and ICG-MB (0) cohorts. This may indicate that MB is equally as sensitive as ICG or the combination, although our study was not specifically designed to establish this. 

To enhance the detection rates of metastases, SLN mapping with pathologic ultrastaging following routine hematoxylin and eosin staining is currently recommended in endometrial carcinoma. Ultrastaging has led to an increased detection rate of low-volume metastases (4.5%) that would otherwise go undetected with routine evaluations [26,27]. It is plausible that our study could have identified more metastases if pathological evaluation with ultrastaging had been performed. Unfortunately, we did not implement it due to a lack of financial resources.

To compare our study’s key findings with others, we conducted an advanced search on PubMed. The main findings of our study and the included studies are provided in Table 6.

Most studies have evaluated SLN staining only in endometrial carcinoma, but some studies have evaluated both endometrial and cervical cancer. Of all the studies, the largest study sample consisted of 204 patients. All studies used either blue dye (BD) or ICG among the included dyes. Blue dyes included MB, Isosulfane blue dye (ISB), or BD without specifying a particular type. When comparing ISB with MB, the research indicated that MB was more cost-effective and equally as effective as ISB, while the use of ISB was associated with a significant number of allergic reactions, some of which were life-threatening [32]. 

Out of all the studies, only Rosenholtz et al. conducted research assessing SLN staining by injecting both dyes simultaneously and evaluating the outcomes in both hemipelvis areas. In all studies, including ours, both the overall detection rate and the bilateral detection rate with ICG were superior to that with the blue dye. Three studies, including ours, evaluated tracer dye combinations. Two out of the three showed an advantage of using ICG-BD, while one demonstrated ICG-BD to be slightly less effective than ICG alone. Taking all the studies into account, the prevailing view is that ICG has an advantage over BD.

Our study demonstrated lower detection rates compared to other studies despite employing the same dyes and injection techniques as other authors. However, these findings emphasize the fact that theoretical detection rates may significantly decrease in a real-world environment. Although our study was randomized and kept the groups similar in size and other population parameters, thus revealing intriguing tendencies, the limited sample size affected the significance of the data, where clearly visible differences were found to be insignificant. As the size of the sample remains the main limitation of this study, we are actively continuing our work to enroll more patients and explore factors associated with achieving better overall and bilateral detection rates. As our study enrolls only early-stage patients, most of them do not undergo full systematic lymphadenectomy; therefore, the sensitivity and negative predictive value of these SLN detection techniques could not be calculated, yet this was not our study goal. The introduction of ultrastaging is one of our future goals, which could benefit patients and the study itself by possibly identifying more micrometastases. It is important to note that these findings are interim and further research will contribute to a more comprehensive understanding of the subject. Moreover, it is essential to consider potential publication bias, where only studies with significant results are published, potentially distorting the overall evidence base in the field. Despite these limitations, our study demonstrates strengths, including appropriate research methodology, reliable data collection techniques, and statistical analysis that confirms previous findings. 

## 5. Conclusions

The combination of ICG-MB tracer dyes in SLN mapping demonstrated superior overall and bilateral detection rates compared to ICG or MB alone in early-stage endometrial cancer, but a significant difference was found only in the MB cohort. ICG, especially in cases where SLN was detected, was the primary tracer in the ICG-MB combination, highlighting the importance of ICG. However, MB proved useful, particularly in cases where ICG stained the entire internal iliac region. This emphasizes the potential complementary role of MB in specific scenarios, proving the choice of the combination of both dyes is superior to using only one. Combining mapping agents can enhance the accuracy of SLN identification and may ensure fewer “empty packets” in initial-stage endometrial cancer without additional risk to the patient.

## Figures and Tables

**Table 1:** Demographic and clinical variables of the patients included in the analysis.

Variable	Global Cohort (99)	MB (35) (a)	ICG (33) (b)	ICG-MB (31) (c)	*p*-Value	
Age (years) (mean (95% CI))	62.9 ± 9.63 (35 to 83)	63.5 ± 10.19 (35 to 83)	62.7 ± 9.19 (36 to 79)	62.4 ± 9.71 (48 to 83)	0.243	ANOVA
BMI (kg/m^2^) (mean (95% CI))	32 ± 6 (18 to 50)	32 ± 6 (23 to 45)	32 ± 6 (21 to 50)	31 ± 6 (18 to 45)	0.4288	ANOVA
Tumor maximum diameter (mm) (mean (95% CI))	27.5 ± 16.8 (1 to 85)	32.8 ± 19.6 (1 to 85)	23.6 ±13.4 (1 to 55)	26.4 ± 16.5 (1 to 65)	0.4482	ANOVA
Histology (*n* (%))					0.38	Fisher’s exact test
Endometrioid adenocarcinoma	88 (89)	32 (91.4)	29 (87.9)	27 (87.1)	0.27	a to b
Clear cell carcinoma	1 (1)	1 (2.9)	0 (0)	0 (0)	0.464	b to c
Serous carcinoma	4 (4)	0 (0)	3 (9.1)	1 (3.2)	0.649	a to c
Mixed adenocarcinoma	6 (6)	2 (5.7)	1 (3.0)	3 (9.7)		
Histologic grade (*n* (%))					0.213	Fisher’s exact test
G1	70 (70.7)	27 (77.2)	19 (57.6)	24 (77.4)	0.131	a to b
G2	17 (17.2)	4 (11.4)	10 (30.3)	3 (9.7)	0.13	b to c
G3	12 (12.1)	4 (11.4)	4 (12.1)	4 (12.9)	1	a to c
Lymphovascular invasion (*n* (%))					0.001	Fisher’s exact test
YES	18 (18.2)	8 (22.9)	10 (30.3)	0 (0)	0.586	a to b
NO	81 (81.8)	27 (77.1)	23 (69.7)	31 (100)	0.001	b to c
					0.005	a to c
FIGO stage (*n* (%))					0.285	Fisher’s exact test
IA	62 (62.6)	21 (60.0)	20 (60.6)	21 (67.7)	0.889	a to b
IB	27 (27.3)	8 (22.8)	9 (27.3)	10 (33.3)	0.351	b to c
II	1 (1)	1 (2.9)	0 (0)	0 (0)	0.089	a to c
IIIA	1 (1)	0 (0)	1 (3.0)	0 (0)		
IIIC	8 (8.1)	5 (14.3)	3 (9.1)	0 (0)		
Type of surgery					0.195	Fisher’s exact test
TH + BSO	14 (14.1)	9 (25.7)	3 (9.1)	2 (6.5)	0.112	a to b
TH + BSO + SLNB	45 (45.5)	12 (34.3)	16 (48.5)	17 (54.8)	0.941	b to c
TH + BSO + uPLn	10 (10.1)	6 (17.2)	2 (6.1)	2 (6.4)	0.112	a to c
TH + BSO + bPLn	19 (19.2)	4 (11.4)	9 (27.2)	6 (19.4)		
TH + BSO + bPLn + AoLn	11 (11.1)	4 (11.4)	3 (9.1)	4 (12.9)		

Abbreviations: TH—total hysterectomy; BSO—bilateral salpingo-oophorectomy; SLNB—sentinel lymph node biopsy; uPLn—unilateral pelvic lymphadenectomy; bPLN—bilateral pelvic lymphadenectomy; AoLn—aortic lymphadenectomy. a—methylene blue group; b—indocyanine green group; c—indocyanine green and methylene blue combination group.

**Table 2:** SLN detection rate.

Variable	Global Cohort (99)	MB (35) (a)	ICG (33) (b)	ICG-MB (31) (c)	*p*-Value	a to b	b to c	a to c
Overall detection rate (*n* (%))	68 (68.7)	19 (54.3)	24 (72.7)	25 (80.6)	0.061	0.137	0.556	0.035
Bilateral detection rate (*n* (%))	38 (38.4)	8 (22.9)	13 (39.4)	17 (54.8)	0.031	0.191	0.316	0.011
Aortic detection rate (*n* (%))	6 (6.1)	1 (2.9)	2 (6.1)	3 (9.7)	0.437	0.608	0.667	0.335

a—methylene blue group; b—indocyanine green group; c—indocyanine green and methylene blue combination group.

**Table 3:** MB dyeing used in combination.

Variable	ICG Used in Combination	MB Used in Combination	*p*-Value
Absence (*n* (%))	6 (19.4)	16 (51.6)	0.004
Unilateral pelvic (*n* (%))	5 (16.1)	9 (29.0)	
Unilateral pelvic + aortic (*n* (%))	1 (3.2)	0 (0)	
Bilateral pelvic (*n* (%))	16 (51.7)	6 (19.4)	
Bilateral pelvic + aortic (*n* (%))	1 (3.2)	0 (0)	
Aortic (*n* (%))	2 (6.4)	0 (0)	

**Table 4:** Lymph node detection sites.

Value	General	MB (a)	ICG (b)	ICG-MB (c)	*p*-Value	
Obturator	29 (26.6)	6 (21.4)	10 (22.7)	13 (35.1)	0.238	Fisher’s exact test
Internal iliac	41 (37.6)	10 (35.7)	19 (43.2)	12 (32.5)	0.19	a to b
External iliac	28 (25.7)	11 (39.3)	7 (15.9)	10 (27.0)	0.207	b to c
Common iliac	4 (3.7)	0 (0)	4 (9.1)	0 (0)	0.667	a to c
Presacral	2 (1.8)	0 (0)	1 (2.3)	1 (2.7)		
Para-aortic	5 (4.6)	1 (3.6)	3 (6.8)	1 (2.7)		
Total	109	28	44	37		

a—methylene blue group; b—indocyanine green group; c—indocyanine green and methylene blue combination group.

**Table 5:** Sentinel lymph node biopsy variables.

Variable	Global Cohort (99)	MB (35) (a)	ICG (33) (b)	ICG-MB (31) (c)	*p*-Value	
SLN drainage, per patient (*n* (%))					0.078	Fisher’s exact test
Absence	31 (31.3)	16 (45.7)	9 (27.3)	6 (19.4)	0.197	a to b
Unilateral pelvic	24(24.2)	11 (31.4)	8 (24.2)	5 (16.1)	0.803	b to c
Unilateral pelvic + aortic	3 (3.0)	0 (0)	2 (6.1)	1 (3.2)	0.008	a to c
Bilateral pelvic	35 (35.5)	7 (20.0)	12 (36.4)	16 (51.7)		
Bilateral pelvic + aortic	3 (3.0)	1 (2.9)	1 (3.0)	1 (3.2)		
Aortic	3 (3.0)	0 (0)	1 (3.0)	2 (6.4)		
SLN per patient (median (IQR))	1 (0 to 8)	1 (0 to 4)	2 (0 to 8)	2 (0 to 7)	0.222	ANOVA
SLN size (mm) (mean (95% CI))	15.1 ± 7.5 (4 to 37)	18.7 ± 9.7 (4 to 37)	13.2 ± 5.2 (6 to 23)	14.1 ± 6.7 (5 to 32)	0.964	ANOVA
SLN mts status per patient (*n* (%))					0.017	Fisher’s exact test
Negative	61 (61.6)	15 (42.9)	21 (63.6)	25 (80.6)	0.239	a to b
Positive	7 (7.1)	4 (11.4)	3 (9.1)	0 (0)	0.148	b to c
Absence of drainage	31 (31.3)	16 (45.7)	9 (27.3)	6 (19.4)	0.003	a to c
Empty node packets (empty packets/total nodes per group) (*n* (%))	4/164 (2.4)	0/34 (0)	4/67 (5.9)	0/63 (0)	0.075	Fisher’s exact test
					0.296	a to b
					0.119	b to c
					1	a to c

a—methylene blue group; b—indocyanine green group; c—indocyanine green and methylene blue combination group.

**Table 6:** The main findings of studies including blue dye and ICG.

Interpretation	Number of SLNs Identified	Primary Disease	Number of Patients	Authors Year
ICG method identified more SLNs than MB; however, the most effective identification occurred when combining methods.	164 SLNs in total The overall DR: 54.3% identified with MB; 72.7% identified with ICG; 80.6% identified with ICG-MB; The bilateral DR was: 22.9% with MB; 39.4% with ICG; 54.8% with ICG-MB.	Endometrial cancer	99	Our study
ICG method identified more SLNs than ISB with no difference in the pathological confirmation of nodal tissue between the two mapping substances.	485 SLNs in total The overall DR: 97% identified with ICG; 95% identified with ICG-ISB; 47% identified with ISB; The bilateral DR was: 32% with ISB; 81% with ICG.	Cervical or endometrial cancer	180	Frumovitz et al. [25] 2018
ICG improved the detection rate of pelvic SLN compared to blue dye.	The overall DR: 96% identified with ICG; 86% identified with BD; The bilateral DR was: 61% with BD; 78% with ICG.	Cervical or endometrial cancer	109	Pölcher et al. [28] 2021
ICG was more effective in detecting SLN compared to ISB.	184 SLNs in total The overall DR: 83% identified with ICG; 64% identified with ISB;	Endometrial cancer	204	Backes et al. [29] 2021
ICG + ISB detected more SLNs and more LN metastases than ISB alone.	The bilateral DR was: 83.9% ICG-ISB; 40% ISB.	Endometrial cancer	200	Holloway et al. [21] 2017
ICG was more effective in the detection rate of hemipelvis SLN compared to blue dye.	The overall DR in hemipelvis: 90.9% identified with ICG; 64.4% identified with BD.	Endometrial cancer	132	Rozenholc et al. [30] 2019
ICG had a significantly higher SLN detection rate than BD in both overall and bilateral detection.	286 SLNs in total The overall DR: 87% identified with ICG; 71% identified with BD; The bilateral DR: 65% identified with ICG; 43% identified with BD.	Endometrial cancer	100	How et al. [31] 2015
SLN mapping using ICG demonstrated higher DR compared to other modalities.	The overall DR: 100% identified with ICG; 89% identified with BD; The bilateral DR: 85% identified with ICG 54% identified with BD.	Cervical or endometrial cancer	163	Buda et al. [17] 2016

Abbreviations: DR—detection rate; SLNs—sentinel lymph nodes; MB—methylene blue; ICG—Indocyanine green; ICG-MB—Indocyanine green and methylene blue combination; ISB—Isosulfane blue dye; BD—blue dye; LN—lymph node.

## Data Availability

Data are currently unavailable due to privacy restrictions, as the research is still ongoing. The data will be shared after the study is completed and the final results are published.
